# Evaluation of prognostic biomarkers in a population-validated Finnish HNSCC patient cohort

**DOI:** 10.1007/s00405-021-06650-7

**Published:** 2021-02-13

**Authors:** J. Routila, I. Leivo, H. Minn, J. Westermarck, Sami Ventelä

**Affiliations:** 1 Turku Bioscience Centre, University of Turku and Åbo Akademi University, Turku, Finland; 2 Department for Otorhinolaryngology, Head and Neck Surgery, University of Turku and Turku University Hospital, Kiinamyllynkatu 4-8, 20521 Turku, Finland; 3 Biomedical Institute, University of Turku, Kiinamyllynkatu 10, 20520 Turku, Finland; 4 FICAN West Cancer Centre, Turku, Finland; 5 Department of Oncology and Radiotherapy, University of Turku and Turku University Hospital, Turku, Finland

**Keywords:** HNSCC, Population-validation, Prognosis, Biomarkers

## Abstract

**Introduction:**

Prognostic biomarkers and novel therapeutic approaches have been slow to emerge in the treatment of head and neck squamous cell carcinoma (HNSCC). In this study, an HNSCC patient cohort is created and performance of putative prognostic biomarkers investigated in a population-validated setting. The overall goal is to develop a novel way to combine biomarker analyses with population-level clinical data on HNSCC patients and thus to improve the carryover of biomarkers into clinical practice.

**Materials and methods:**

To avoid selection biases in retrospective study design, all HNSCC patients were identified and corresponding clinical data were collected from the Southwest Finland geographical area. A particular emphasis was laid on avoiding potential biases in sample selection for immunohistochemical staining analyses. Staining results were evaluated for potential prognostic resolution.

**Results:**

After comprehensive evaluation, the patient cohort was found to be representative of the background population in terms of clinical characteristics such as patient age and TNM stage distribution. A negligible drop-out of 1.3% (6/476) was observed during the first follow-up year. By immunohistochemical analysis, the role of previously implicated HNSCC biomarkers (p53, EGFR, p16, CIP2A, Oct4, MET, and NDFIP1) was investigated.

**Discussion:**

Our exceptionally representative patient material supports the use of population validation to improve the applicability of results to real-life situations. The failure of the putative prognostic biomarkers emphasizes the need for controlling bias in retrospective studies, especially in the heterogenous tumor environment of HNSCC. The resolution of simple prognostic examination is unlikely to be sufficient to identify biomarkers for clinical practice of HNSCC.

**Supplementary Information:**

The online version contains supplementary material available at 10.1007/s00405-021-06650-7.

## Introduction

Head and neck squamous cell carcinomas (HNSCC) compose a behaviorally diverse field of cancers united by their common localization to the head and neck regions [1, 2]. Clinical problems such as early metastatic behavior and serial recurrences due to field cancerization are frequently encountered. Especially intriguing phenomena are the unexpected aggressiveness of small tumors and, in a favorable way, the surprising treatment response of some large tumors. The current therapy stratification of HNSCC is based on the overall state of the patient and clinical observations about the tumor [3, 4].

The site and extent of the tumor do not, however, have a decisive effect on patient prognosis [5, 6]. Attempts to explain clinical diversity of HNSCC by genetical and molecular analysis have thus far proven unsuccessful, leaving the determination of patient prognosis uncertain. A multitude of biomarkers has been suggested, with little success in translating findings to clinical practice [7]. The enthusiastically awaited inclusion of p16/HPV in the staging of oropharyngeal HNSCC has not met all expectations [8]. Some reasons to lack of success may be found in the uneven inclusion of patients to especially small retrospective patient cohorts, bias in inclusion criteria, and poor definition of clinical questions to be tackled [7, 9].

Northern European healthcare system offers an intriguing prospect for unbiased patient sampling, because cancer patients in need of oncological treatment are referred to regional tertiary centers independent of insurance or socioeconomic status of the patients. In addition, based on EUROCARE-5 data, the results of head and neck cancer treatment in Nordic countries and especially in Finland are remarkably superior to other regions in Europe [10].

In this study, a population-based cohort of all new HNSCC patients treated between 2005 and 2010 in Southwest Finland region, covering one sixth of Finland’s population, was collected. This cohort of HNSCC patients corresponds to the real-life patient succession treated at our institute. Tumor samples were retrieved, sampling bias analyzed, and a panel of immunohistochemical biomarkers analyzed.

Thus, we re-evaluated the real-life capability of a panel of immunohistochemical biomarkers to prognosticate patient 5-year overall survival (OS), when identified clinical prognostic variables are taken into account. All of these biomarkers have previously been reported to function as prognostic markers in HNSCC. The biomarkers included loss of tumor suppressor p53 expression associated with p53 mutations, that are the most often encountered mutations in HNSCC associated with metastatic behavior and radio resistance [11]. EGFR overexpression has been the focus of intense study in HNSCC, as EGFR inhibitors are available [12]. p16 has a clinical application as oropharyngeal cancer prognosticator [13, 14]. CIP2A is an mTOR and MYC-associated inhibitor of tumor suppressor protein phosphatase 2A [15]. MET and Oct4 are associated with a stemness phenotype [16, 17] and NDFIP1 was listed in the top three unfavorable HNSCC biomarker in Protein Atlas database [18].

## Materials and methods

### Primary HNSCC patient cohort

The HNSCC patient cohort was formed by identifying and including all patients treated for new HNSCC in Turku University Hospital (TUH) region in 2005–2010. Tumors were staged according to TNM criteria applicable at the time of diagnosis. Treatment protocols were decided in a multidisciplinary Tumor Board for head and neck cancer. OS was defined from end-of-treatment to end-of-follow-up or death. Age-standardized OS were calculated using International Cancer Survival Standards for weighting.

The usage of human tissue samples was approved by the Finnish national authority for medicolegal affairs (V/39706/2019), regional ethics committee of University of Turku (51/1803/2017) and Auria biobank scientific board (AB19-6863). Patient formalin-fixed, paraffin-embedded (FFPE) samples were acquired from pathology archives through Auria Biobank. Final TMA blocks of duplicate 0.6 mm cores were made in TMA Grand Master (3D Histech) according to annotations on scanned HE slides. Samples of normal liver were included in each block for orientation.

### Immunohistochemistry (IHC)

FFPE blocks were cut into 6 um sections. CIP2A IHC was carried out after protocol optimization in Ventana BenchMark XT staining automate (Ventana Medical Systems, Inc) using mouse monoclonal anti-CIP2A antibody (1:25, 2G10-3B5, sc-80659, SantaCruz). p16, p53, and EGFR IHC were carried out in Ventana in clinical pathology laboratory. Oct4 IHC was performed as previously described with anti-Oct4 antibody sc-5279 (1:200 mouse monoclonal, Santa Cruz Biotechnology) [17]. NDFIP1 immunohistochemistry was carried out with anti-NDFIP1 antibody HPA009682 (1:1000 rabbit polyclonal, Atlas Antibodies). MET stainings were performed as previously reported [16].

Immunohistochemical stainings were analyzed by two authors independently, and differences were discussed until consensus was reached. p53 staining was analyzed using the established 3-tier system. Cytoplasmic/membraneous EGFR, MET, and CIP2A expression were scored semiquantitatively based on intensity of the staining on a scale of 1–3. Nuclear Oct4 was scored positive, when a subpopulation of strong positive nuclei was present. p16 immunostaining was regarded positive, when at least 70% of cells demonstrated strong nuclear and cytoplasmic staining intensity. Nuclear NDFIP1 staining was regarded positive when strong, uniform nuclear staining was present. For all statistical analyses, dichotomous cutoffs were applied.

### Statistical analysis

Patient data and staining results were entered into SPSS 24 software (SPSS, IBM). For Cox hazards models, the proportionality of hazards was testing using log-minus-log plotting and plotting Schoenfeld residuals against survival time, when appropriate. For all multivariable analysis, stepwise approach with backward LR method was applied, if not otherwise indicated, with *p* value limits for inclusion and exclusion at 0.05 and 0.10, respectively. For Kaplan–Meier survival estimation, significance was analyzed using log-rank method. To test prognostic potential of biomarkers, their combinations and their interactions, Cox regression was used by first entering the prognostic clinicopathological variables and in another block the biomarker combinations. *p* values of less than 0.05 were considered significant.

## Results

### Southwest Finland regional cohort corresponds with Nordic EUROCARE-5 population

**Fig. 1:**
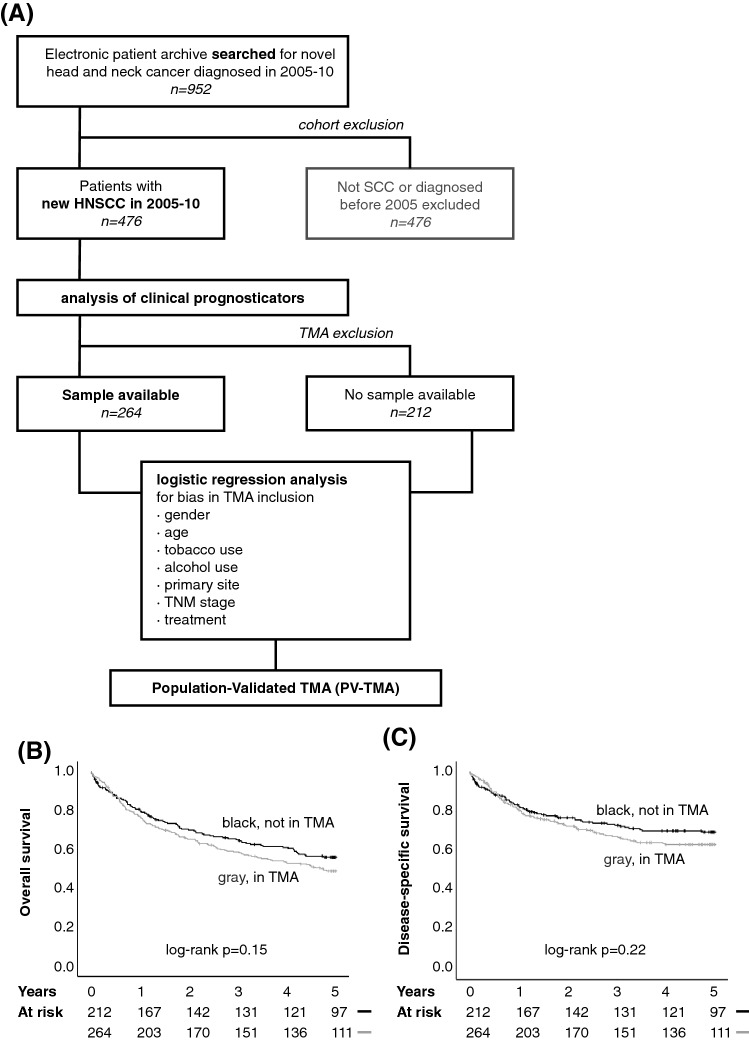
**a** Principle of the population-validated TMA. First, a background population was screened for comprehensive inclusion of all patients treated for HNSCC in Southwest Finland during the time period of 2005–2010. This background population was used to assess clinical prognostic factors. All available samples were included in TMA. The representativeness of the TMA was analyzed with logistic regression analysis for multiple variables. After the representativeness was confirmed, the TMA is considered a population-validated TMA (PV-TMA). **b** Overall survival, and **c** disease-specific survival of the patients included in PV-TMA was slightly lower than of patients not included in PV-TMA. In multivariable analysis, there was no difference in survival

An electronic database screen was made to include all HNSCC patients treated in Southwest Finland region during years 2005–2010 (Fig. 1a). Altogether 952 patients’ records were accessed. After initial evaluation, the final cohort included 476 patients diagnosed and treated for new HNSCC tumor (Table 1). Two-hundred and thirty-two patients (49%) were diagnosed with early stage HNSCC, 164 patients (34%) had nodal metastasis at presentation, and five patients (1.1%) were diagnosed with distant metastasis. Only 1.3% (6/476) of patients were lost during the first year of follow-up.

**Table 1:** Clinicopathological variables of the patient cohort. Univariate (left panels) and multivariable (right panels) survival analysis of HNSCC cohort

	Total		Survival effect		Total		Survival effect	
	*n*	*%*	HR (95% CI)	*p*	*n*	*%*	HR (95% CI)	*p*
Gender								
Male	325	68	1.03 (0.78–1.37)	0.84	325	68	not included	–
Female	151	32	1	–	151	32		
Age at diagnosis								
< 65	236	50	1.02 (1.01–1.03) /year	< 0.001	236	50	1.04 (1.02–1.05) / yr	< 0.001
> 65	240	50	–		240	50	–	
Smoking status								
Current smoker	202	42	1.31 (0.99–1.74)	0.063	202	42	1.19 (0.80–1.78)	0.39
Former smoker	73	15	0.93 (0.61–1.41)	0.73	73	15	0.89 (0.57–1.39)	0.61
Non-smoker	201	42	1	–	201	42	1	–
Alcohol consumption								
Yes	139	29	0.69 (0.52–0.91)	0.008	139	29	1.45 (1.02–2.06)	0.037
No	337	71	1	–	337	71	1	–
Primary tumor site								
Oral cavity	226	47	1	–	226	47	1	–
Oropharynx	89	19	0.86 (0.59–1.26)	0.86	89	19	0.69 (0.46–1.05)	0.086
Larynx	105	22	1.24 (0.90–1.73)	0.19	105	22	1.03 (0.71–1.49)	0.88
Hypopharynx	20	4	2.65 (1.51–4.63)	0.001	20	4	1.61 (0.88–2.96)	0.13
Other	36	8	1.08 (0.65–1.78)	0.77	36	8	1.15 (0.67–1.97)	0.61
T class								
T0-2	311	65	0.32 (0.24–0.41)	< 0.001	311	65	0.27 (0.17–0.44)	< 0.001
T3-4	165	35	1	–	165	35	1	–
N class								
N0	312	66	0.67 (0.51–0.88)	0.003	312	66	0.54 (0.36–0.78)	0.001
N +	164	34	1	–	164	34	1	–
Stage								
0–II	232	49	0.46 (0.35–0.60)	< 0.001	232	49	1.41 (0.77–2.58)	0.26
III–IV	244	51	1	–	244	51	1	–
Recidive in 5 years								
Yes	137	29	5.34 (3.92–7.27)	< 0.001	137	29	not included	–
No	289	61	1	–	289	61	–	–
No curative treatment	49	10	30.07 (20.06–45.08)	< 0.001	49	10	–	–
Living at 5 years								
Yes	253	53	–	–	253	53	not included	–
No, died of HNSCC	150	32	–	–	150	32	–	–
No, died of other cause	73	15	–	–	73	15	–	–
Surgical treatment								
No surgery	141	30	1	–	141	30	1	–
Local operation	282	59	0.59 (0.45–0.76)	< 0.001	282	59	0.74 (0.55–0.98)	0.038
Neck dissection	173	36	0.86 (0.66–1.14)	0.29	173	36	0.73 (0.53–1.00)	0.049
Treatment type								
Surgery only	172	36	1	–	172	36	1	–
RT only	51	11	2.71 (1.78–4.12)	< 0.001	51	11	2.12 (1.26–3.57)	0.005
CRT only	75	16	1.57 (1.05–2.36)	0.028	75	16	0.81 (0.46–1.43)	0.47
RT + surgery	46	10	1.97 (1.24–3.11)	0.004	46	10	1.27 (0.77–2.07)	0.40
CRT + surgery	116	24	1.16 (0.80–1.70)	0.43	116	24	0.74 (0.44–1.23)	0.25
No treatment	15	3	15.75 (8.82–28.17)	< 0.001	15	3	5.80 (2.96–11.38)	< 0.001

Results from Cox proportional hazards model regression. In multivariable modeling, treatment effects were analyzed by entering the clinical prognostic variables (separated by a horizontal line.)

**Fig. 2:**
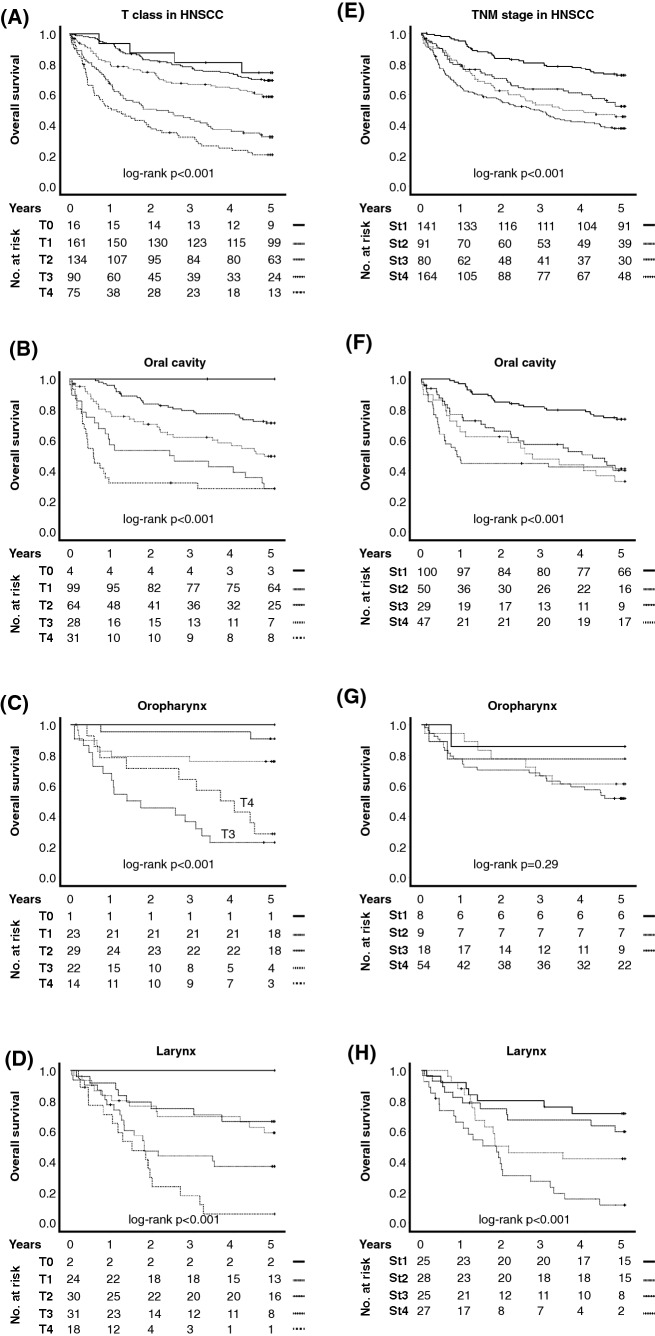
Overall survival was highly affected by tumor T class in both **a** HNSCC overall and the three main subsites, **b** oral cavity, **c** oropharynx, and **d** larynx. **e**–**h** TNM stage was an inferior prognosticator as compared to tumor T class in HNSCC overall and the three main subsites, especially in oropharynx, where the prognostic resolution was virtually non-existent. In oral cancer, TNM stage offered minimal prognostic resolution between stage 2 and stage 3

OS was influenced by previously acknowledged risk factors: patient age, advanced T class, nodal positivity, and alcohol use (Table 1). Interestingly, T class proved to be a superior prognosticator than TNM stage in all major subsites of HNSCC (Fig. 2a–h and Table 1). However, inadequate prognostic resolution between T1 and T2 as well as T3 and T4, respectively, was noted, especially in laryngeal cancer (Fig. 2d). Thus, for multivariable analysis, T class was divided dichotomously in T0-2 vs T3-4, providing a highly significant prognostic stratification (Table 1; HR 0.27, 95% CI 0.17–0.44, *p* < 0.001). While the primary tumor site had no decisive impact on patient OS, inclusion of primary tumor site in the following multivariable models was deemed appropriate.

One-hundred and seventy-two patients (36%) were given only surgical treatment (Table 1). Ninety-seven and 191 patients were treated with radiotherapy or chemoradiotherapy, respectively. Fifteen patients were offered no treatment. In a multivariable model fitting age at diagnosis, primary tumor site, T class, nodal status and alcohol consumption, no treatment type proved clearly superior with regard to OS impact, although surgical treatment was associated with a statistically significant improvement in prognosis.

Survival data were compared to results of EUROCARE-5 study (summarized in Table 2). In comparison to general Finnish, Northern European, and whole European average head and neck cancer patient survival, the observed survival rates in Southwest Finland region were higher especially in elderly patients and hypopharyngeal cancer.

**Table 2:** Survival rates in TUH HNSCC patient cohort compared with Eurocare-5 data for Northern Europe

	Oral cavity		Larynx		Oropharynx		Hypopharynx		Total	
	HNSCC	Eurocare	HNSCC	Eurocare	HNSCC	Eurocare	HNSCC	Eurocare	HNSCC	Eurocare
OAS 5-years	56%	–	48%	–	70%	–	30%	–	53%	–
DSS 5-years	71%	–	69%	–	65%	–	40%	–	68%	–
ICSS 5-year observed survival rate	58%	43%	50%	52%	57%	41%	36%	17%	53%	41%
ICSS 5-year relative survival rate	–	50%	–	62%	–	46%	–	19%	–	46%

Eurocare-5 data accessed at https://w3.iss.it/site/EU5Results/

### Construction of representative population-validated tissue microarray (PV-TMA)

Altogether 264 patients’ tumor samples were available for TMA (Fig. 1a). A thorough analysis of TMA construction biases was carried out (Table 3). Compared to clinical data of the background population, HNSCC patients treated in Southwest Finland region in 2005–2010, the established PV-TMA was shown to be representative in terms of age distribution, tobacco and alcohol exposure and especially TNM class, whereas uneven site distribution was observed.

**Table 3:** Univariate (left panels) and multivariable (right panels) analysis of TMA inclusion bias

	Total		TMA patients		TMA inclusion		TMA patients		TMA inclusion	
	*n*	%	*n*	*%*	OR (95% CI)	*p*	*n*	%	OR (95% CI)	*p*
Gender										
Male	325	68	164	62	0.52 (0.35–0.78)	0.001	164	62	0.56 (0.36–0.88)	0.011
Female	151	32	100	38	1	–	100	38	1	–
Age at diagnosis										
< 65	236	50	137	52	1.23 (0.86–1.77)	0.26	137	52	Not included	
> 65	240	50	127	48	1	–	127	48		
Smoker										
> 20 pack years	225	47	115	44	0.72 (0.50–1.03)	0.071	115	44	NS	
< 20 pack years	251	53	149	56	1	–	149	56		
Alcohol consumption										
Yes	139	29	78	30	0.96 (0.65–1.43)	0.85	78	30	Not included	
No	337	71	186	70	1	–	186	70		
Primary tumor site										
Oral cavity	226	47	137	52	1	–	137	52	1	–
Oropharynx	89	19	64	24	1.66 (0.98–2.84)	0.062	64	24	2.34 (1.21–4.54)	0.012
Larynx	105	22	35	13	0.33 (0.20–0.53)	< 0.001	35	13	0.68 (0.37–1.24)	0.21
Hypopharynx	20	4	11	4	0.79 (0.32–1.99)	0.62	11	4	1.64 (0.58–4.63)	0.35
Other	36	8	17	6	0.58 (0.29–1.18)	0.13	17	6	0.93 (0.42–2.03)	0.85
T class										
T0-2	311	65	173	66	1.02 (0.70–1.49)	0.92	173	66	Not included	
T3-4	165	35	91	34	1	–	91	34		
N class										
N0	312	66	157	59	0.54 (0.37–0.80)	0.002	157	59	NS	
N +	164	34	107	41	1	–	107	41		
Stage										
0–II	232	49	118	45	0.70 (0.48–1.00)	0.049	118	45	NS	
III–IV	244	51	146	55	1	–	146	55		
Recidive in 5 yrs										
Yes	137	29	84	32	1.40 (0.93–2.12)	0.11	84	32	Not Included	
No	289	61	152	58	1	–	152	58		
No curative treatment	49	10	28	11	1.20 (0.65–2.21)	0.56	28	11		
Living at 5 years										
Yes	253	53	131	50	0.72 (0.48–1.08)	0.11	131	50	Not Included	
No, died of HNSCC	150	32	90	34	1	–	90	34		
No, died of other cause	73	15	43	16	0.96 (0.54–1.69)	0.88	43	16		
Surgical treatment										
No surgery	141	30		0	1	–	55	21	1	–
Local operation	282	59	174	66	1.88 (1.30–2.73)	0.001	174	66	1.75 (1.05–2.94)	0.033
Neck dissection	173	36	125	47	3.10 (2.07–4.63)	< 0.001	125	47	2.30 (1.49–3.56)	< 0,001
Treatment type										
Surgery only	172	36	92	35	1	–	92	35	NS	
RT only	51	11	20	8	0.56 (0.30–1.06)	0.075	20	8		
CRT only	75	16	30	11	0.58 (0.33–1.01)	0.052	30	11		
RT + surgery	46	10	34	13	2.46 (1.20–5.08)	0.015	34	13		
CRT + surgery	116	24	82	31	2.10 (1.27–3.46)	0.004	82	31		
no treatment	15	3	5	2	0.44 (0.14–1.33)	0.14	5	2		

Results from logistic regression modeling

Importantly, TMA inclusion was not a significant predictor of 5-year OS or disease-specific survival in neither univariate analysis nor in multivariable survival model fitting for established clinical risk factors (Fig. 1b, c). In conclusion, the PV-TMA constructed for this work can be considered to be well representative of HNSCC patients treated in the region of Southwest Finland in 2005–2010.

### Analysis of representative HNSCC patient TMA demonstrates poor performance of putative biomarkers for prognostication

**Fig. 3:**
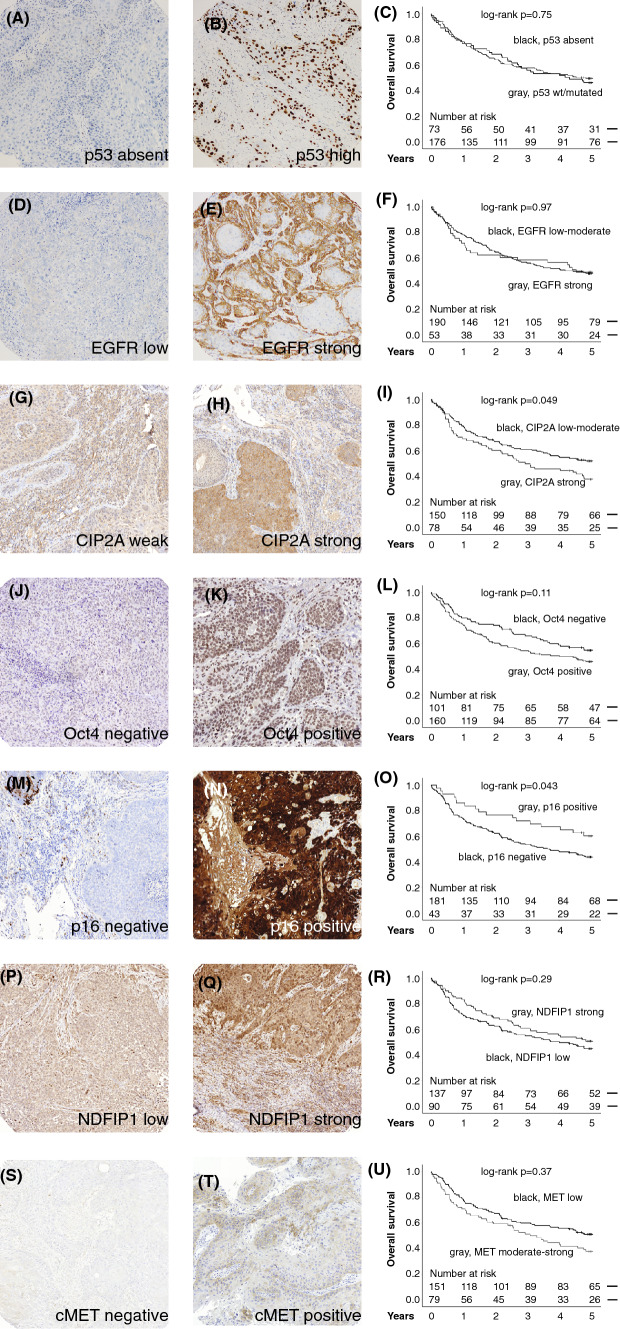
Representative immunohistochemical stains and prognostic trends (estimates using Kaplan–Meier method and log-rank method for significance) of the investigated biomarkers in HNSCC. **a**–**c** p53, **d**–**f** EGFR, **g**–**i** CIP2A, **j**-**l** Oct4, **m**–**o** p16, **p**–**r** NDFIP1, **s**–**u** MET

Using this exceptionally representative PV-TMA material, we analyzed the prognostication capability of multiple biomarkers—p53, EGFR, p16, CIP2A, MET, Oct4, and NDFIP1—previously shown to function as prognostic markers in HNSCC (Fig. 3). The prognostic information of CIP2A and p16 reached significance in univariate analysis (Fig. 3i, o, respectively). However, regardless of the hypothesis-based selection of the candidate biomarkers and their previous association with poor prognosis in HNSCC, none of the biomarkers showed significant prognostic value in multivariable analysis using PV-TMA material (Table 4).

**Table 4:** Prognostic performance of investigated biomarker staining intensities

	Total		5-yr survival		Survival analysis	
	*n*	%	alive,* n*	%	HR	*p*
p53						
Absent	73	29	34	47	0.91 (0.62–1.36)	0.65
wt or high	176	71	89	51	1	–
EGFR						
Low-moderate	190	78	93	49	1.27 (0.82–1.97)	0.29
Strong	53	22	26	49	1	–
CIP2A						
Low-moderate	150	66	79	53	1.20 (0.81–1.76)	0.37
High	78	34	30	38	1	–
Oct4						
Negative	101	39	56	55	0.73 (0.50–1.07)	0.11
Positive	160	61	75	47	1	–
p16						
Negative	181	81	81	45	0.91 (0.68–1.22)	0.54
Positive	43	19	26	60	1	–
NDFIP1						
Negative	137	60	63	46	1.18 (0.81–1.73)	0.40
Positive	90	40	46	51	1	–
cMET						
Low	151	66	76	50	0.80 (0.55–1.15)	0.22
Moderate-high	79	34	38	48	1	–

Staining distributions, survival rates, and results of multivariable survival analysis (Cox proportional hazards model controlling for age, T class, nodal status and alcohol use)

Further, the possible prognostication value of the biomarkers for oral cavity, oropharyngeal, or laryngeal cancer patients was further investigated using a multivariable model entering the above identified clinical prognosticators. None of the investigated biomarkers provided statistically significant prognostic information in the three main subsites of HNSCC (Supplementary Table 1). Furthermore, no combination or interaction of the investigated biomarkers could not provide significant prognostic potential in multivariable survival regression, when clinical prognostic variables were included in the models (data not shown).

## Discussion

Our study demonstrates, that in a non-biased HNSCC patient population treated with optimal results, the putative biomarkers failed to offer significant prognostic information. In order to improve retrospective as well as future prospective studies, a population-based analysis should be mandatory to appreciate the potential biases in patient selection. Further, the recent failures of significant prospective drug trials in HNSCC [19–21] suggest that optimization of retrospective studies is an underappreciated step in discovery of biomarkers for patient treatment stratification.

This study emphasizes the need for thorough exploration of inclusion bias, since some exclusion of patients due to loss of samples and inadequate sample size is unavoidable. In our patient cohort, this is achieved by analysis of the population giving rise to the TMA cohort, the Southwest Finland HNSCC patients from 2005 to 2010. The statistical analysis reveals that our PV-TMA is an exceptionally representative and unbiased study environment for retrospective analysis of biomarkers. Population-validation approach thus improves the robustness and reliability of data analysis.

High risk of bias is present in patient inclusion to both retrospective and prospective cohorts [22, 23]. Inclusion biases include unequal recruitment of patients with different socioeconomic status or limited insurance coverage, supposedly having a poor prognosis, and on the other hand patients with small tumors with good prognosis. Moreover, variance in the given cancer treatments between different hospitals, and between individual clinicians can also be a confounding factor in the analysis of treatment outcomes. Clinical validation of our patient cohort is made possible by the referral system in Northern Europe, leading to an unbiased, institutional patient population, which serves as a representative cross-section of the regional population. Thus, this dataset represents the real-life patient succession observed in the clinic and is, in this respect, superior to recruited prospective cohorts. Furthermore, loss to follow-up is virtually non-existent due to the Nordic public health care system and electronic databases.

Particularly good head and neck cancer treatment results in Nordic countries increases the interest of this dataset [10]. Interestingly, in our regional data, the Southwest Finland patient prognosis was even better than in Finnish EUROCARE-5 data. This may be due to more wide-spread use of cisplatin radiosensitization and, most importantly, the long-standing multi-disciplinary tumor board practice, guaranteeing optimized protocols, meticulous treatment planning, and impartial response monitoring. Of special clinical interest is also the superior prognostic resolution afforded by T class in comparison to complete TNM stage. However, the observed 34% survival rate of T1-2 patients provides rationale for biomarker-based prognostication.

Particularly interesting are our results when putative biomarkers with auspicious publication history for prognostication of HNSCC were tested in PV-TMA. Importantly, we failed to recognize significant prognostic factors, when clinical prognosticators were taken into account, either in the patient material as a whole or in any major subsite. Surprisingly, no combination or interaction of biomarkers proved useful in prognostication of our patient material. More complex statistical analysis used in previous studies to create prognostic biomarker panels [24, 25] could not be applied in this study, concentrating in an unselected patient population. Despite the disappointing failure of the biomarkers, our approach highlights the value of unbiased cross-sectional regional control of patient inclusion in biomarker discovery.

Immunohistochemistry for p16 is the only clinically approved biomarker for HNSCC and is applied in oropharyngeal cancer staging. In our study, p16 was a surprisingly poor prognosticator of HNSCC patients’ OS, in contrast to earlier reports [14, 26, 27]. Whether this is attributable to better overall prognosis of HPV-negative patients or the widespread use of cisplatin radiosensitization, remains an intriguing question. The failure of recent p16 deintensification trials seems, however, to demonstrate a need for better understanding of the role of p16 in both radio- and chemoradioresistance [8, 20]. Thus, our finding cautions against p16-based deintensification with regard to current treatment guidelines in Finland.

The main strength of this study is the impartial inclusion of all HNSCC patients treated in our regional referral center. Thus, the patient cohort is representative of the real-life population encountered in the routine clinical practice, increasing the applicability of our results to clinical decision-making. Despite the crucial representativeness of our patient cohort, there are weaknesses in this study as well. The patient number remains relatively low, especially in site-specific analysis. Further, the patient numbers do not readily allow for more complex statistical approaches, such as multivariable analysis of biomarker combinations and more detailed analysis of staining cut-offs, including integration of data on subcellular localization changes.

In conclusion, we demonstrate the value of population-validation methodology for retrospective biomarker studies, and wish to emphasize the need for population level evaluation for inclusion biases. Impartial cancer patient selection, comprehensive patient registers available for researchers, and exceptionally good cancer treatment outcomes demonstrate optimal possibilities for retrospective analysis of biomarkers. Similar approach should be applied for the design of future prospective trials in molecularly diverse cancers.

## Supplementary Information

Below is the link to the electronic supplementary material.

Supplementary file1 (DOCX 14 KB)
