# Supplementary material for: Evaluation of prognostic biomarkers in a population-validated Finnish HNSCC patient cohort
Source: Eur Arch Otorhinolaryngol. 2021 Feb 13;278(11):4575–85. doi: 10.1007/s00405-021-06650-7 (PMC8486716; doi:10.1007/s00405-021-06650-7)
Supplement: Supplementary file 1 — Supplementary file1 (DOCX 14 KB) [file 405_2021_6650_MOESM1_ESM.docx]

| Supplemental Table 1. **Results of multivariable site-specific survival analysis of the investigated biomarkers.** | | | | | | | |
| --- | --- | --- | --- | --- | --- | --- | --- |
|  |  | **Oral cavity** | | **Oropharynx** | | **Larynx** | |
|  |  | *HR* | *p* | *HR* | *p* | *HR* | *p* |
| **p53** |  |  |  |  |  |  |  |
|  | *absent* | 0.86 (0.50-1.47) | 0.57 | 0.66 (0.24-1.83) | 0.43 | 0.55 (0.19-1.60) | 0.27 |
|  | *wt or high* | 1 | - | 1 | - | 1 | - |
| **EGFR** |  |  |  |  |  |  |  |
|  | *low-moderate* | 1.08 (0.59-1.95) | 0.81 | 0.57 (0.19-1.77) | 0.33 | 0.90 (0.28-2.89) | 0.86 |
|  | *strong* | 1 | - | 1 | - | 1 | - |
| **CIP2A** |  |  |  |  |  |  |  |
|  | *low-moderate* | 0.71 (0.40-1.27) | 0.25 | 0.86 (0.38-1.95) | 0.72 | 0.61 (0.18-2.06) | 0.43 |
|  | *high* | 1 |  | 1 | - | 1 | - |
| **Oct4** |  |  |  |  |  |  |  |
|  | *negative* | 0.77 (0.45-1.31) | 0.33 | 1.42 (0.57-3.58) | 0.45 | 0.31 (0.06-1.48) | 0.14 |
|  | *positive* | 1 | - | 1 | - | 1 | - |
| **p16** |  |  |  |  |  |  |  |
|  | *negative* | 2.72 (0.63-11.8) | 0.18 | 0.88 (0.35-2.22) | 0.79 | 0.45 (0.05-4.42) | 0.50 |
|  | *positive* | 1 | - | 1 | - | 1 | - |
| **NDFIP1** |  |  |  |  |  |  |  |
|  | *negative* | 1.63 (0.86-3.08) | 0.13 | 0.91 (0.42-1.96) | 0.81 | 0.57 (0.16-2.06) | 0.39 |
|  | *positive* | 1 | - | 1 | - | 1 | - |
| **cMET** |  |  |  |  |  |  |  |
|  | *low* | 0.93 (0.54-1.58) | 0.78 | 0.73 (0.33-1.64) | 0.45 | 1.41 (0.30-6.54) | 0.66 |
|  | *moderate-high* | 1 | - | 1 | - | 1 | - |
|  |  |  |  |  |  |  |  |
